# 2′-*O*-Methyl RNA/Ethylene-Bridged Nucleic Acid Chimera Antisense Oligonucleotides to Induce *Dystrophin* Exon 45 Skipping

**DOI:** 10.3390/genes8020067

**Published:** 2017-02-10

**Authors:** Tomoko Lee, Hiroyuki Awano, Mariko Yagi, Masaaki Matsumoto, Nobuaki Watanabe, Ryoya Goda, Makoto Koizumi, Yasuhiro Takeshima, Masafumi Matsuo

**Affiliations:** 1 Department of Pediatrics, Hyogo College of Medicine, Nishinomiya 6638501, Japan; leeleetomo@me.com (T.L.); ytake@hyo-med.ac.jp (Y.T.); 2 Department of Pediatrics, Kobe University Graduate School of Medicine, Kobe 6500017, Japan; awahiro@med.kobe-u.ac.jp (H.A.); mmatsu@med.kobe-u.ac.jp (M.M.); 3 Nikoniko House Medical and Welfare Center, Kobe 6511102, Japan; marikoyagi.sgt@gmail.com; 4 Drug Metabolism & Pharmacokinetics Research Laboratories, Daiichi Sankyo Co., Ltd., Tokyo 1408710, Japan; watanabe.nobuaki.nc@daiichisankyo.co.jp (N.W.); goda.ryoya.u4@daiichisankyo.co.jp (R.G.); 5 Modality Research Laboratories, Daiichi Sankyo Co., Ltd., Tokyo 1408710, Japan; koizumi.makoto.h7@daiichisankyo.co.jp; 6 Department of Physical Therapy, Faculty of Rehabilitation, Kobe Gakuin University, Kobe 6512180, Japan

**Keywords:** 2′-*O*,4′-*C*-ethylene-bridged nucleic acid, ENA, antisense oligonucleotide, exon skipping therapy, Duchenne muscular dystrophy

## Abstract

Duchenne muscular dystrophy (DMD) is a fatal muscle-wasting disease characterized by dystrophin deficiency from mutations in the *dystrophin* gene. Antisense oligonucleotide (AO)-mediated exon skipping targets restoration of the *dystrophin* reading frame to allow production of an internally deleted dystrophin protein with functional benefit for DMD patients who have out-of-frame deletions. After accelerated US approval of eteplirsen (Exondys 51), which targets *dystrophin* exon 51 for skipping, efforts are now focused on targeting other exons. For improved clinical benefits, this strategy requires more studies of the delivery method and modification of nucleic acids. We studied a nucleotide with a 2′-*O*,4′-*C*-ethylene-bridged nucleic acid (ENA), which shows high nuclease resistance and high affinity for complementary RNA strands. Here, we describe the process of developing a 2′-*O*-methyl RNA(2′-OMeRNA)/ENA chimera AO to induce *dystrophin* exon 45 skipping. One 18-mer 2′-OMeRNA/ENA chimera (AO85) had the most potent activity for inducing exon 45 skipping in cultured myotubes. AO85 was administered to *mdx* mice without significant side effects. AO85 transfection into cultured myotubes from 13 DMD patients induced exon 45 skipping in all samples at different levels and dystrophin expression in 11 patients. These results suggest the possible efficacy of AO-mediated exon skipping changes in individual patients and highlight the 2′-OMeRNA/ENA chimera AO as a potential fundamental treatment for DMD.

## 1. Introduction

Duchenne muscular dystrophy (DMD) (OMIM 310200) is the most common inherited muscle disease in childhood, affecting more than one in every 3500 live-born boys. DMD is caused by mutations in the *dystrophin* gene and characterized by dystrophin deficiency in muscles. It is a progressive muscle-wasting disease and usually fatal in the third or fourth decades of life. Although general medical treatments and physical therapy have slowly increased life expectancy [1,2], establishment of a fundamental treatment that leads to dystrophin expression has long been the ultimate goal in DMD research.

To recover dystrophin expression in DMD, gene replacement therapy is a logically appropriate strategy, but more studies are needed to form the basis for clinical translation [3,4]. As an alternative, induction of exon skipping with antisense oligonucleotides (AOs) that convert out-of-frame *dystrophin* mRNA into in-frame sequences has been proposed [5]. Exonic deletion mutations in the *dystrophin* gene cause not only DMD but also Becker muscular dystrophy (BMD), a milder progressive muscle-wasting disease. A translational reading frame rule explains the difference between DMD and BMD [6]: Out-of-frame exon deletion mutations create premature stop codons that result in the more severe DMD phenotype because of a total lack of the dystrophin protein. In contrast, exon deletion mutations that maintain the original reading frame in the mRNA lead to the milder BMD phenotype because a mutated, but still functional, dystrophin protein can be expressed from the mRNA. Therefore, exon skipping allowing one exon to be omitted from the mutated *dystrophin* mRNA can be used to restore the reading frame and produce the internally deleted dystrophin [7]. Compared with gene replacement therapy, the benefits of exon skipping with AOs include the ability to use the endogenous gene and straightforward chemical synthesis of AOs.

We demonstrated the first clinical study of exon skipping therapy using a phosphorothioate-oligonucleotide against *dystrophin* exon 19 and showed successful induction of exon skipping and dystrophin expression in skeletal muscle in a DMD patient (Patient 0) [8]. However, the effectiveness was not enough to improve his motor function. This study has demonstrated a proof of concept as an AO-mediated exon skipping to treat DMD [9]. Subsequently, in order to develop successful AO-mediated exon skipping therapy, various kinds of modified AOs have been chemically synthesized, with some of the most prominent being phosphorodiamidate morpholino oligonucleotides (PMOs), 2′-*O*-methyl (2′-OMe) AOs, locked nucleic acid (LNA), peptide nucleic acid (PNA), and tricycle-DNA (tcDNA) [10,11]. Furthermore, chimera AOs such as anhydrohexitol nucleic acid (HNA)/2′-OMe phosphorothioate (2′OMePS), cyclohexenyl nucleic acid (CeNA)/2′OMePS, altritol nucleic acid (ANA)/2′OMePS, and morpholino nucleic acid (MNA)/2′OMePS have been investigated [12,13]. Among these AOs, two AOs comprising different monomers—PMOs [14] and 2′-OMePS [15]—were developed to induce skipping of *dystrophin* exon 51 and have undergone clinical trials. These trials yielded good results, enabling improvements in ambulation in DMD patients [16,17,18,19]. One PMO oligonucleotide, eteplirsen-mediated (Sarepta Therapeutics Inc., Cambridge, MA, USA) exon skipping therapy led to a slower rate of decline in ambulation compared to historical controls [18]. After a long heated discussion [20], eteplirsen has received accelerated approval from the US Food and Drug Administration (FDA) [21]. At the same time FDA stated that a clinical benefit of eteplirsen has not been established [22]. There is controversy as to whether it is really beneficial [23,24,25].

Now, AO-mediated exon skipping therapy is reaching the final stage to provide a treatment for DMD patients. Efforts are focused on targeting other exons [4,26]. However, it is true that this therapy can stand further improvement in modification of nucleic acids, their delivery method, and evaluation methods [10]. To improve clinical benefits, more studies are needed.

After establishing the proof of concept as AO-mediated exon skipping therapy, we have continuously developed AOs comprising a modified nucleotide with an ethylene bridge between oxygen at the 2′-position and carbon at the 4′-position of ribose (2′-*O*,4′-*C*-ethylene-bridged nucleic acid; ENA^®^, Daiichi Sankyo Co., Ltd., Tokyo, Japan), which is highly nuclease resistant and shows a high affinity for complementary RNA strands [27]. Here, we describe the progress in developing the 2′-*O*-methyl RNA(2′-OMeRNA)/ENA chimera AO to induce *dystrophin* exon 45 skipping.

## 2. Identification of AO for Exon 45 Skipping

### 2.1. A Modified Nucleic Acid of ENA^®^

**Figure 1:**
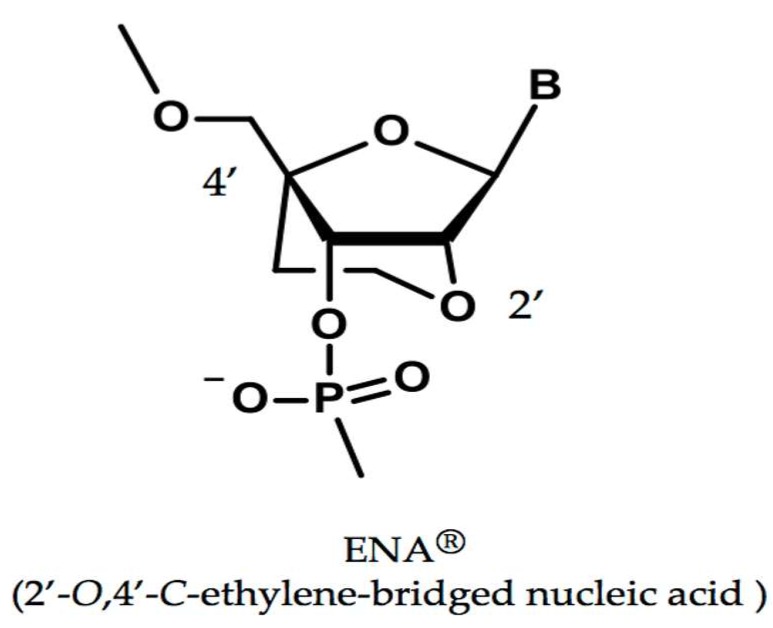
Structure of 2′-*O*,4′-*C*-ethylene-bridged nucleic acid (ENA). ENA has an ethylene bridge between the oxygen at the 2′-position and carbon at the 4′-position of ribose. B indicates base.

To improve the efficacy of nucleic acids, several modified nucleic acids have been created to provide higher nuclease resistance and high affinity for complementary sequences. ENA with an ethylene bridge between the oxygen and carbon of ribose (Figure 1) is thermodynamically stable and highly nuclease resistant [28,29] and has a high affinity for complementary RNA strands [30,31]. In fact, the ability of an AO consisting of the 2′-OMeRNA/ENA chimera to induce exon 19 skipping is more than 40 times stronger than that of the conventional phosphorothioate backbone oligonucleotides, making it a promising candidate for use as a low-toxicity, high-affinity oligonucleotide in the long-term treatment of DMD [32]. Furthermore, a 2′-OMeRNA/ENA chimera against *dystrophin* exon 41 encoding a nonsense mutation has been shown to induce efficient skipping of the mutated exon 41 [33]. Therefore, we decided to use 2′-OMeRNA/ENA chimera oligonucleotides for exon skipping.

### 2.2. 2′-OMeRNA/ENA Chimera AO to Skip Exon 45

**Figure 2:**
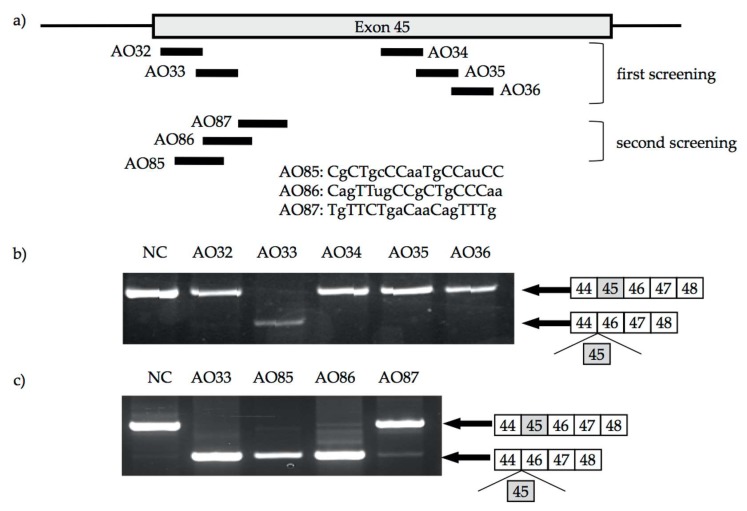
Identification of the best antisense oligonucleotide (AO) to induce exon 45 skipping. 2′-OMeRNA/ENA chimera AOs were designed to cover splicing enhancers in exon 45 and synthesized. A portion of 200 nM of each AO was transfected into myotubes. The resulting *dystrophin* mRNA was analyzed by RT-PCR amplification. The procedure of this analysis has been described in detail by Takeshima et al. [34]. (**a**) Locations of AOs are schematically described. Box and bars indicate exon 45 and flanking introns, respectively. Bars represent AOs. The AOs shown in the upper and the lower halves were used in the first and second screening steps, respectively. Nucleotide sequences of AO85, AO86, and AO87 were described (bottom). Upper and lower-case letters represent ENA-modified residues and 2′-*O*-methyl RNA, respectively; (**b**) Electropherograms of RT-PCR products. A dystrophin cDNA fragment from exons 44 to 48 was PCR amplified. PCR amplification revealed two products in AO33-treated cells (AO33) and one product in other treatments (AO32, AO34, AO35, and AO36). The additional smaller product in AO33-treated cells lacked exon 45, indicating exon 45 skipping. Lane NC represents non-transfected control (NC). The exons in the amplified products are shown schematically on the right, and the shaded box represents the AO target exon; (**c**) Electropherograms of RT-PCR amplification products of the second screening step for AO-induced dystrophin exon 45 skipping. All three AOs induced exon 45 skipping, but only AO85 induced exon 45 skipping perfectly.

Exon skipping therapy is mutation specific. To develop the broad therapeutic applicability of exon skipping therapy, we focused on induction of exon 45 skipping because it can be applied for treatment in approximately 9% of DMD patients [19,34]. The best AO to induce exon 45 skipping was identified by a trial-and-error procedure as described previously [35]. 2′-OMeRNA/ENA chimera AOs consisting of 2′-*O*-methyl RNA with phosphodiester backbone, and ENA residues (at cytosines and thymines or at both the 5’ and 3’-ends) were employed. As the first screening for the identification of AOs against exon 45, five 15-mer AOs (AO32–36) to cover the splicing enhancer sequence of exon 45 were designed (Figure 2a) and examined for their ability to induce exon 45 skipping. Among five AOs, only one, AO33, induced exon 45 skipping (Figure 2b). To identify a more suitable AO, another set of 18-mer AOs (AO85–87) neighboring AO33 was designed (Figure 2a). Although all three AOs induced exon 45 skipping, only AO85 did so perfectly (Figure 2c). Therefore, AO85 was selected as the optimal AO for exon 45 skipping.

### 2.3. Characterization of AO85

**Figure 3:**
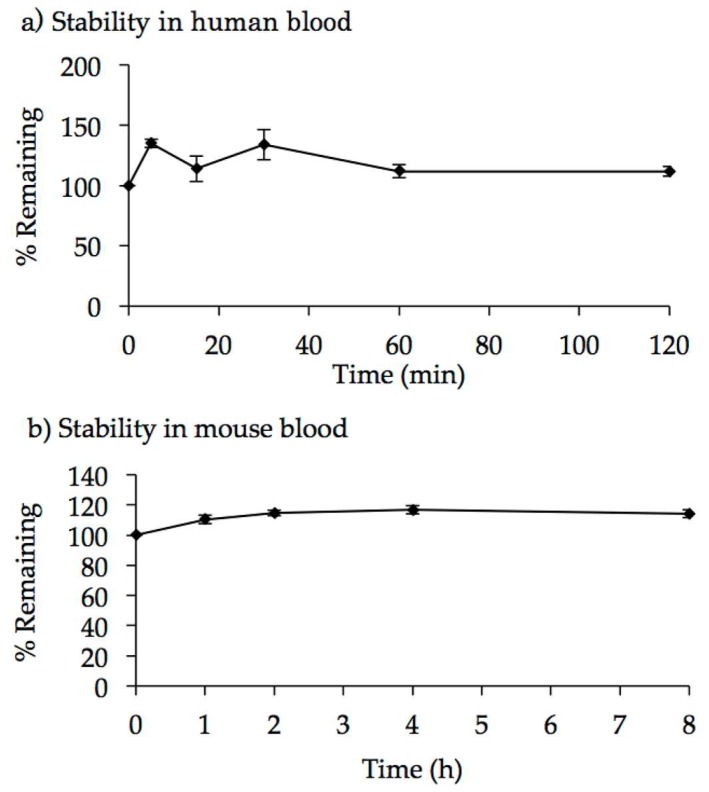
Stability of AO85 in human and mouse blood. Human blood was collected from three healthy volunteers, who had received no drugs for more than one week before blood sampling, with written informed consent. A 5 μL aliquot of AO85 solution (1.0 mg/mL) was added to the freshly obtained human individual blood (2.5 mL each, *n* = 3) at a final concentration of 2.0 μg/mL. Aliquots of the blood incubation mixture were taken at the time points of 0, 5, 15, 30, 60, and 120 min after incubation at 37 °C. Mouse blood was obtained from male mice (C57BL/6NCrlCrlj, *n* = 42, Charles River Laboratories, Yokohama, Japan) under isoflurane anesthesia. A 20 μL aliquot of AO85 solution (1.0 mg/mL) was added to the freshly obtained pooled mouse blood (10 mL) at a final concentration of 2.0 μg/mL, which was divided into three portions prior to incubation. Aliquots of the blood incubation mixture were taken at the time points of 0, 1, 2, 4, and 8 h after incubation at 37 °C. An aliquot (100 μL) of each blood sample following centrifugation at 15,000 rpm at 4 °C for 5 min was mixed with 100 μL aqueous methanol containing an internal standard (IS) compound, followed by filtration using the Amicon Ultra filter (YM-50) and concentration using the Amicon Ultra filter device (YM-3). The samples were analyzed by LC-MS/MS system (HPLC: Ultimate, MS: TSQ Vantage, Thermo Fisher Scientific Inc., Waltham, MA, USA), and peak area ratio (AO85/IS) was determined. Then, the data at each time point were expressed as stability %, with peak area ratio at 0 min being 100%. (**a**) Stability is shown as % remaining at each time point. In human blood, the peak area ratio of AO85/IS remained stable even for 120 min; (**b**) In mouse blood, the peak area ratio of AO85/IS remained stable even for 8 h.

The half-maximum effective concentration of AO85 was determined in a cell-free splicing system and found to be 58 nM [36]. To determine the stability of AO85, it was incubated with human and mouse blood. The mass peak corresponding specifically to the unchanged form of the full-length AO85 was monitored by LC-MS/MS system. The peak area ratio of AO85 to the internal standard (IS) remained stable for 2 and 8 h in human and mouse blood, respectively (Figure 3), demonstrating that AO85 remained as the full-length unchanged form over the time course of the incubation in human and mouse blood. Consistent with previous studies [28,29] on other ENA-AOs, AO85 was found to be stable in blood.

In our previous reports AO85 at 400 nM caused a 30% decrease in cell viability in MTT assay using fibroblasts [37]. To examine the toxicity of AO85, we performed a single dose-escalation study using *mdx* mice, an animal model of DMD (Table 1). The *mdx* mice received intravenous administration of AO85 at doses of 0, 5, 15, and 50 mg/kg. They were observed for 14 days, and no abnormalities, including in body weight change, were found. Autopsy examination at Day 14 revealed no apparent abnormal pathological change in *mdx* mice treated with 0, 5, and 15 mg/kg. However, four of five mice treated with 50 mg/kg of AO85 showed renal enlargement, and two of five showed cysts on the renal surface. These findings suggested renal damage caused by high-dose AO85.

Next, we performed a repeated-administration toxicity study using *mdx* mice (Table 2). Intravenous AO85 injection at doses of 0, 0.5, and 5.0 mg/kg was repeated four times at one-week intervals in five *mdx* mice. Over four weeks, one mouse receiving 0 mg/kg died because of varicella; no other animals died. No abnormal finding was observed regarding the general condition, weight change, food intake, blood examination, and pathological findings. This result suggested that repeated administration of AO85 is safe.

## 3. Efficacy of AO85 in Myotubes from DMD Patients

### 3.1. AO85 Efficacy in Myotubes from a DMD Patient with a Deletion Mutation of Exon 46–51

**Figure 4:**
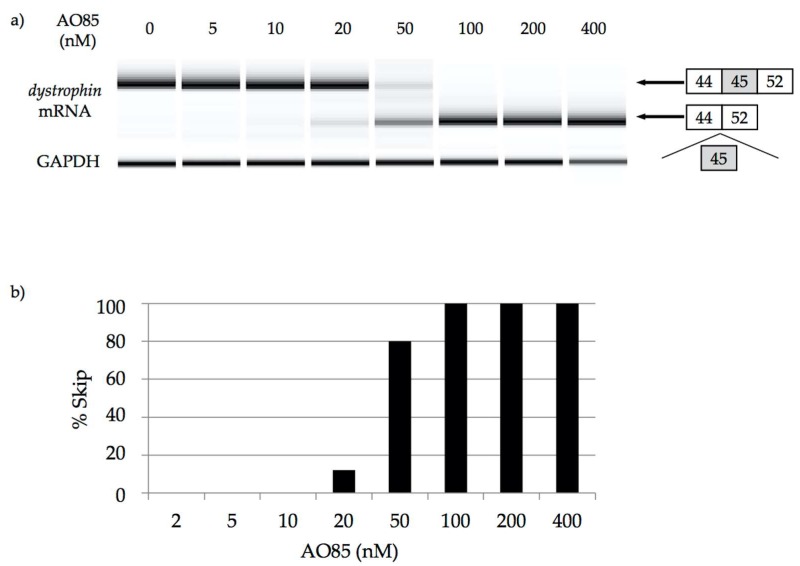
Induction of exon 45 skipping with AO85 in Duchenne muscular dystrophy (DMD) myotubes. Preparation of myotubes from a DMD patient with a deletion mutation of exons 46–51 and examination of exon skipping by AO85 transfection were conducted as described previously [32]. AO85 was added into cultured medium at concentrations of 0, 5, 10, 20, 50, 100, 200, and 400 nM. cDNA was prepared from 1 μg of total RNA. The *dystrophin* mRNA was analyzed by RT-PCR amplification. (**a**) The result of RT-PCR amplification. At the concentration over 20 nM, RT-PCR amplification revealed shorter product lacking exon 45, which was not observed at the concentration lower than 10 nM. Only shorter product was observed at the concentration over 100 nM; (**b**) The exon-45-skipping efficacy by AO85. Skipping efficiencies were determined from gel images by quantifying the skipped products with a DNA 1000 LabChip Kit on an Agilent 2100 Bioanalyzer (Agilent Technologies, Santa Clara, CA, USA). At the concentration over 100 nM, 100% of the *dystrophin* mRNA showed exon 45 skipping.

To confirm the dose-dependent effect of AO85, it was transfected into cultured myotubes from a DMD patient with a deletion mutation of exon 46–51. RT-PCR amplification of mRNA in cultured myotubes treated at the concentration over 20 nM revealed a shorter product lacking exon 45, which was not observed in cultured myotubes at the concentrations lower than 10 nM (Figure 4a). At the concentrations over 100 nM, 100% of the *dystrophin* mRNA showed exon 45 skipping (Figure 4b).

**Figure 5:**
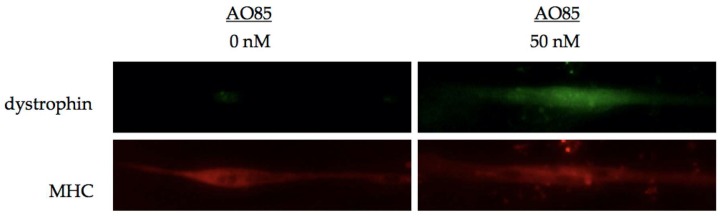
Dystrophin expression in cultured DMD myotubes by AO85 transfection. AO85 was transfected into the myotubes prepared as described in Figure 4 at a concentration of 50 nM. Seven days after the transfection, treated myotubes were harvested. After fixation with cold methanol, the fixed myotubes were incubated with polyclonal anti-dystrophin antibody (targeted to the C-terminal region; ab15277, Abcam, Cambridge, UK) and anti-myosin heavy chain (MHC) antibody (sc 101334, Santa Cruz Biotechnology, Dallas, TX, USA). The secondary antibodies were conjugated with Alexa Fluor 488 (Abcam) for dystrophin and Alexa Fluor 568 (Abcam) for the MHC. Immunohistochemical staining of dystrophin and MHC are shown. Before treatment, dystrophin was negative. After the transfection of AO85 (50 nM), myotubes were dystrophin positive, as was MHC staining. A quantitative assessment of dystrophin expression was not performed. Studies on DMD patients in this article were done after obtaining informed consent, in addition to approval from the local ethics committee.

Next, we analyzed dystrophin expression in cultured myotubes from the same patient with a deletion mutation of exons 46–51 (Figure 5). Before treatment with AO, immunohistochemical staining showed no dystrophin signal. However, at 7 days after transfection with AO85 (50 nM), immunohistochemical staining disclosed antibody-reacting materials in myotubes treated with AO85. These results revealed that AO85 transfection could induce exon 45 skipping and lead to dystrophin production in DMD myotubes.

### 3.2. AO85 Efficacy in Myotubes from 13 DMD Patients

We examined the ability of AO85 to induce exon 45 skipping and dystrophin expression in myotubes from 13 DMD patients carrying six different deletion mutations, such as deletions of exon 44, 46–47, 46–48, 46–49, 46–51, or 46–53. In each case, exon 45 skipping was induced by AO85, as expected; however, the skipping efficiency was different from patient to patient (Table 3). In myotubes from three patients with the exon 46–51 deletion, more than 80% of the *dystrophin* mRNA was exon skipping product while less than 43% of it was in myotubes carrying the exon 46–47 deletion. Dystrophin expression was seen in all but two patients. Unexpectedly, in myotubes with the exon 46–51 deletion, dystrophin expression was not apparent even though 82% showed exon 45 skipping in the mRNA. On the other hand, in myotubes with exon 46–47 deletion, dystrophin expression was apparent in spite of a low rate of exon 45 skipping in the mRNA.

## 4. Discussion

Here, we have shown the process of developing the 2′-OMeRNA/ENA chimera AO against exon 45 and identified one suitable candidate, AO85. AO85 was effective in inducing exon 45 skipping and proved to be safe. These findings paved the way for clinical trials. In one clinical study, in fact, AO85 was shown safe and effective in increasing six-minute walk distance when it was injected intravenously at a dose of 0.5mg/kg/week [38]. Currently, one 2′-OMeRNA/ENA chimera AO against dystrophin exon 45 (DS-5141b) is under clinical trial in Japan (JapicCTI No: 153072, http://www.clinicaltrials.jp/user/cte_main.jsp). For induction of exon 45 skipping, another AO consisting of PMO (SRP-4045, Sarepta Therapeutics) is now in clinical trial (https://clinicaltrials.gov).

Previous clinical trials of AO-mediated exon skipping have suggested a risk of renal impairment such as proteinuria [4]. Although single high-dose AO85 administration induced slight renal change, repeated administration of AO85 at the appropriate clinical dose (0.5 mg/kg) revealed no toxicity. AO85 was considered not toxic to the kidney.

Theoretically, exon 45 skipping efficiency should be equal in all affordable patients. However, our current investigation showed the surprising result of differences in exon skipping efficiency among the patients. Even in same-age patients, efficiency varied, suggesting that age is not a determining factor in efficacy differences. We also found a difference in exon skipping efficacy between patients with the 46–51 deletion (82%–100%) and those with the 46–47 deletion (30%–43%). This difference suggested a relation between the efficiency of exon skipping and the breakpoint sequence of the deletion mutation [39], which requires further study for confirmation.

In-frame exon 45–46 deletions result in a severe DMD phenotype [40]. Although these deletions are the outliers from the reading frame rule, the mechanism that produces the outlier is not known. Currently, the discrepancy between levels of exon skipping and dystrophin expression is not explainable. A limitation of this study was that quantitative evaluation was not performed, and more research is needed to clarify the mechanism that results in the discrepancy between exon skipping and dystrophin expression.

The cost of eteplirsen treatment was estimated $300,000–$400,000 in a year for one patient [22]. To reduce the cost, it is important to develop more efficient AO by either improving drug delivery [41] or using other modified nucleotides. In our case we employed ENA. In the current scheme the dose is only 0.5 mg/kg [37], while 30–50 mg/kg in eteplirsen [16]. Therefore, it is expected that the cost for treatment would be reduced using 2′-OMeRNA/ENA chimera. Efficient targeting of eteplirsen to cardiac muscle remains significant challenges [42]. Our result suggests that the delivery to cardiac muscle of 2′-OMeRNA/ENA chimera AO should be evaluated.

## 5. Conclusions

One 2′-OMeRNA/ENA chimera AO that induces exon 45 skipping was identified and shown to be non-toxic and effective. Although a discrepancy in levels between exon skipping and dystrophin expression was observed among samples, we expect that AO85 can be applied for AO-mediated exon skipping therapy as a fundamental treatment for DMD.

## Figures and Tables

**Table 1:** Dose escalation administration of AO85 in *mdx* mice.

AO85 (mg/kg)	Number of Deaths (Case)	General Condition	Pathological Findings (at Day 14)
0	0	Good	Normal
5	0	Good	Normal
15	0	Good	Normal
50	0	Good	Renal enlargement (4/5 cases) Cyst on renal surface (2/5 cases)

Five mice were administered the same concentration of AO85 at four different levels and observed for 14 days.

**Table 2:** Repeated administration of AO85 into *mdx* mice.

AO85 (mg/kg)	Deaths	General Condition	Laboratory Examination	Pathological Findings (at Day 28)
0	1	Good	Normal	Normal
0.5	0	Good	Normal	Normal
5	0	Good	Normal	Normal

Four times at one-week intervals, five mice were administered the same concentration of AO85 at three different levels.

**Table 3:** AO85-mediated exon 45 skipping and dystrophin expression in 13 DMD patients.

No.	Age	Deleted Exon	Exon-45-Skipped mRNA (%)	Dystrophin Immunostaining
1	5	44	65	Positive
2	5		70	Positive
3	5	46–47	43	Positive
4	5		30	Positive
5	10		40	Positive
6	6	46–48	40	Positive
7	8	46–49	65	Positive
8	5		51	Positive
9	5	46–51	100	Positive
10	5		100	Positive
11	6		82	Negative
12	6	46–53	65	Positive
13	3		45	Negative

Myotubes were prepared from 13 DMD patients and transfected with 100 nM of AO85. The percentages of exon-45-skipped mRNA among total mRNA were calculated as described in Figure 4. Dystrophin immunostaining was evaluated as described in Figure 5 and judged to be positive or negative.
